# Fracture Behavior of Steel-Fiber-Reinforced High-Strength Self-Compacting Concrete: A Digital Image Correlation Analysis

**DOI:** 10.3390/ma18153631

**Published:** 2025-08-01

**Authors:** Maoliang Zhang, Junpeng Chen, Junxia Liu, Huiling Yin, Yan Ma, Fei Yang

**Affiliations:** 1 Henan Building Materials Research and Design Institute Co., Ltd., Zhengzhou 450002, China; zhml82@163.com (M.Z.); huiling2000_yin@163.com (H.Y.); myan0721@163.com (Y.M.); 2 Henan Academy of Sciences, Zhengzhou 450046, China; 3 School of Intelligent Construction and Civil Engineering, Zhongyuan University of Technology, Zhengzhou 450007, China; chenjunpeng93@163.com

**Keywords:** fracture mechanical properties, digital image correlation technology, high-strength self-compacting concrete, fracture toughness

## Abstract

In this study, steel fibers were used to improve the mechanical properties of high-strength self-compacting concrete (HSSCC), and its effect on the fracture mechanical properties was investigated by a three-point bending test with notched beams. Coupled with the digital image correlation (DIC) technique, the fracture process of steel-fiber-reinforced HSSCC was analyzed to elucidate the reinforcing and fracture-resisting mechanisms of steel fibers. The results indicate that the compressive strength and flexural strength of HSSCC cured for 28 days exhibited an initial decrease and then an enhancement as the volume fraction (*V_f_*) of steel fibers increased, whereas the flexural-to-compressive ratio linearly increased. All of them reached their maximum of 110.5 MPa, 11.8 MPa, and 1/9 at 1.2 vol% steel fibers, respectively. Steel fibers significantly improved the peak load (F_P_), peak opening displacement (CMOD_P_), fracture toughness (*K_IC_*), and fracture energy (*G_F_*) of HSSCC. Compared with HSSCC without steel fibers (HSSCC-0), the *F_P_*, *K_IC_*, CMOD_P_, and *G_F_* of HSSCC with 1.2 vol% (HSSCC-1.2) increased by 23.5%, 45.4%, 11.1 times, and 20.1 times, respectively. The horizontal displacement and horizontal strain of steel-fiber-reinforced HSSCC both increased significantly with an increasing *V_f_*. HSSCC-0 experienced unstable fracture without the occurrence of a fracture process zone during the whole fracture damage, whereas the fracture process zone formed at the notched beam tip of HSSCC-1.2 at its initial loading stage and further extended upward in the beams of high-strength self-compacting concrete with a 0.6% volume fraction of steel fibers and HSSCC-1.2 as the load approaches and reaches the peak.

## 1. Introduction

Self-compacting concrete (SCC) exhibits good flowability, uniformity, and stability, all of which give it the ability to completely fill a template under its own gravity. Thus, SCC is widely utilized in complex shapes, dense reinforcements, and thin-walled and fair-faced concrete structures. With the development of complex structural projects such as high-rise buildings, large-span bridges, harbor terminals, and ocean platforms, the requirements for the load-bearing capacity of structural materials are increasing, and the research and applications of high-strength self-compacting concrete (HSSCC) have attracted wide attention [1]. However, HSSCC offers lower bearing and deformation performance under bending loads, especially with respect to its brittleness in the load-softening stage, which greatly restricts its application in engineering structures subjected to wind, earthquakes, impacts, and other loads [2,3].

Steel fibers delay the formation and expansion of microcracks inside high-strength concrete through crack-arresting and crack-reinforcing effects so as to improve its bending and tensile properties and fracture toughness [4]. The deformation performance and toughness of high-strength concrete under various loads were correspondingly improved with an increasing volume fraction of steel fibers [5,6]. A suitable amount of uniformly dispersed steel fibers not only improved the ductility of SCC but also drastically increased its fracture mechanical properties [7,8]. The results of Magbool et al. [9] showed that the fracture energy of high-strength concrete increased by 16 times with the incorporation of 1.0 vol.% steel fibers in comparison to the benchmark group. Yoo et al. [10] demonstrated that the flexural strength, flexural capacity, and post-peak ductility of high-strength concrete increased significantly when the volume fraction of steel fibers exceeded 1.0%, and its fracture energy increased with an increasing volume fraction of steel fibers. Muhammed et al. [11] concluded that, when the mass ratio of steel fibers in cement-based materials reaches 30%, the fracture energy increases by up to 102.2 times. Meng et al. [12] indicated that the energy absorption capacity of steel-fiber-reinforced concrete was closely related to the volume fraction of fibers, and the toughness of steel-fiber-reinforced concrete with different fiber volume fractions could be predicted. Steel fibers effectively inhibited the expansion of cracks in the SCC, and then, the bore parts of the tensile force in the following cracking process increased energy consumption and improved the fracture mechanical properties correspondingly [13].

The digital image correlation (DIC) method, a non-destructive monitoring technique, has been extensively utilized in research fields related to crack initiation and overall damage of concrete [14,15]. Xie et al. [16] monitored the damage evolution of basalt-fiber-reinforced concrete beams during the damage process using the DIC method and explored the effect of basalt fibers on crack extension. Liu et al. [17] verified that high-strength concrete under dynamic tensile stresses exhibited multiple cracking through the fiber-bridging effect using the DIC technique. Dehghani et al. [18] found that shape-memory alloy fibers conferred high-strength concrete with excellent efficacy in healing the cracking process and a more uniform strain distribution using the DIC methodology. The above studies verified the feasibility and reliability of the DIC method when applied to areas related to concrete crack formation and extension.

Building components are subjected to various loads during their long-term service and inevitably generate cracks and defects. Under tensile stress, the bearing capacity and deformation performance of cracked components directly affect the stability of building components. In light of the above findings, based on the preparation of steel-fiber-reinforced HSSCC, its fracture mechanical properties were investigated through a three-point bending test with notched beams. This study clarified the influence of steel-fiber volume fractions (*V_f_*) on mechanical properties, peak loads (*F_P_*), fracture toughness (*K_IC_*), critical crack mouth opening displacement (CMOD_P_), and fracture energy (*G_F_*), as well as the characteristics of the force (*F*)–crack mouth opening displacement (CMOD) curve. Furthermore, the horizontal displacement clouds and horizontal strain clouds of the notched beams were obtained by the DIC method to describe crack propagation behavior during the fracture process of HSSCC. In this study, DIC technique was employed to depict the damage and failure mechanisms of HSSCC at the mesoscopic scale, which effectively addressed the disconnection between fracture performance and the mesoscopic crack evolution behavior. As a result, it provides fundamental data for evaluating the safety of components containing cracks.

## 2. Experimental Materials and Methods

### 2.1. Raw Materials

Grade 52.5 ordinary Portland cement was utilized to ensure the high-strength characteristics of concrete, and the technical indexes of which are listed in Table 1. The slag had a specific surface area of 628 m^2^/kg, a density of 2.93 g/cm^3^, and an activity index of 109%, and its chemical composition is detailed in Table 2. The silica fume contained 92.2% SiO_2_, along with the specific surface area and activity index being 21 m^2^/g and 112%, respectively. Aggregates included gravel and river sand. The coarse aggregate was 5~16 mm continuous graded gravel with an apparent density of 2.58 g/cm^3^. The river sand exhibited a fineness modulus of 2.90 and an apparent density of 2.60 g/cm^3^. Tap water was utilized as the mixing water. A polycarboxylate superplasticizer, LY-2, was employed to ensure the workability requirements of self-compacting concrete. The superplasticizer provided a 30% water reduction rate and simultaneously compensated for an increase in water demand caused by slag and silica fume. The technical specifications of the corrugated-type steel fibers are summarized in Table 3.

### 2.2. Preparation of Steel-Fiber-Reinforced HSSCC

#### 2.2.1. Mixture of Steel-Fiber-Reinforced HSSCC

The mixture of steel-fiber-reinforced HSSCC is presented in Table 4. The water-to-binder ratio (W/B) and sand ratio (*β_s_*) were 0.32 and 0.43, respectively. The volume fraction of steel fibers was set at 0, 0.3%, 0.6%, 0.9%, and 1.2%, with fibers replacing sand and gravel in equal volume proportions. The dosage of water reducer was adjusted to ensure the workability of steel-fiber-reinforced HSSCC.

#### 2.2.2. Testing Methods for Physical and Mechanical Properties

The compressive and flexural strength were determined in accordance with the “Standard for Test Methods of Mechanical Properties of Ordinary Concrete” (GB/T 50081-2019) [19]. The workability was characterized by slump extension, J-ring extension, and T_500_, tested according to Chinese standard for “Technical specification for application of self-compacting concrete” (JGJ/T 283-2012) [20]. A 150 mm cube was prepared for the compressive strength of HSSCC, and a prism of 100 mm × 100 mm × 400 mm was employed for flexural strength testing, with each group consisting of three specimens.

### 2.3. Methods of Fracture Testing and DIC Image Collection

#### 2.3.1. Specimen Preparation

**Figure 1:**
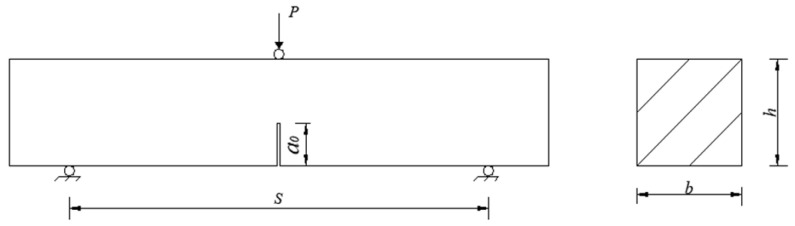
Notched three-point bending beams and its dimensions.

The fracture behaviors of steel-fiber-reinforced HSSCC were characterized by a three-point bending test with the notched beam following the RILEM testing standard [21]. The specimen dimensions and loading method are depicted in Figure 1. The width (*b*) and height (*h*) of the specimen were both 100 mm, the total length (*l*) was 515 mm, the span (*S*) was set to 400 mm, the precast notch depth (a_0_) at mid-span was 40 mm, and the notch width was 3 mm. Specimens cured for 28 days were used in fracture experiments, with three specimens in each group, and were air-dried for 2 days before testing. To facilitate the acquisition DIC images and capture deformation data for all calculation points within the observation area, the flatter molded side of the specimen was selected for scattering surface preparation. The specific preparation process was as follows: A layer of matte white paint was uniformly sprayed as a background on the selected flatter side. After allowing the paint to dry completely, black paint was spattered onto the observation area to create a high-contrast artificial scattering spot.

#### 2.3.2. Fracture Performance Test and DIC Image Collection

**Figure 2:**
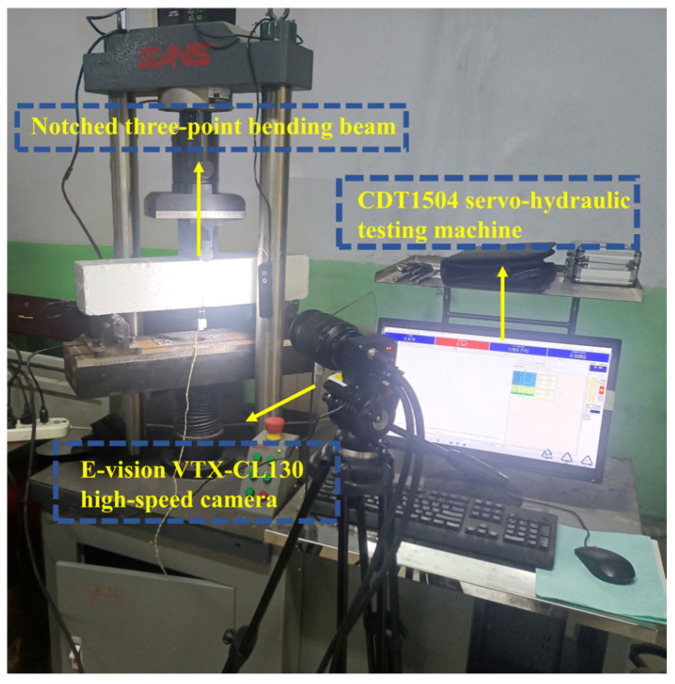
Fracture test site coordinated with DIC technology.

The mass of specimens was weighed with an accuracy of 1 g before conducting the three-point bending test. The distance between two supports was calibrated for each loading to minimize the effects of span errors. Knife-edge steel plates were attached to both sides of the precast notch to fix the clip-on extensometer, which was used for measuring crack mouth opening displacement (CMOD). The tests were conducted on a CDT1504 servo-hydraulic testing machine manufactured by MTS Systems (China) Co., Ltd. in Shanghai, China, under displacement control at a loading rate of 0.05 mm/min. Figure 2 presents the site image of the notched three-point bending beam test synchronized with DIC image collection. The mid-span load (*F*) and deflection (*δ*) were recorded using the built-in data acquisition system of the testing machine, while CMOD was recorded by a clip-type extensometer. The F-CMOD curve was plotted based on the collected load (*F*) and crack mouth opening displacement (CMOD) values.

In order to achieve precise displacement tracking, an artificial speckle pattern was applied on the beam surface, consisting of a matte white paint background and matte black paint speckles. Image acquisition was performed using an E-vision VTX-CL130 high-speed camera manufactured by Shanghai E-vision Optoelectronic Co., Ltd. in Shanghai, China. Before the experiment, the bracket and focal length of the camera lens were adjusted to ensure clear image capture with direct frontal alignment to the observation area. Images of the scattering surfaces were captured at 1 to 2 frames/s during loading. The digital image correlation method grounded in the Newton–Raphson method and the particle swarm optimization algorithm were employed for the image data analysis to obtain two-dimensional contour plots [22].

### 2.4. Fracture Performance Evaluation Method

According to fracture mechanics theory, fracture toughness and fracture energy are two important indexes for assessing the fracture performance of concrete, and both possess significant theoretical and practical implications.

#### 2.4.1. Fracture Toughness

The fracture toughness (*K_IC_*) refers to the stress intensity factor corresponding to the unstable crack extension under elastic–plastic conditions, which reflects the resistance of concrete to crack expansion. In the case of the notched beam three-point bending test, *K_IC_* was calculated according to Formulas (1) and (2) recommended by ASTM.

(1) $$K_{\mathrm{IC}}=\frac{P_{\max}S}{bh^{3/2}}f(a/h)$$

(2) $$f\left(a/h\right)=2.9{\left(a/h\right)}^{1/2}-4.6{\left(a/h\right)}^{3/2}+21.8{\left(a/h\right)}^{5/2}-37.6{\left(a/h\right)}^{7/2}+38.7{\left(a/h\right)}^{9/2}$$

where $P_{max}$ is the maximum load in the three-point bending test, kN. *S* is the span, mm. *b* and *h* represent the width and height of the specimens, respectively, mm. a denotes the critical effective crack length of the concrete specimens. For plain HSSCC, a was equivalent to a_0_, mm. For steel-fiber-reinforced groups, a was calculated by Formulas (3) and (4), where *δ_m_* and *δ_t_* were measured by an extensometer [7].

(3) $$e=\sqrt{{\left(\frac{a_{0}}{\delta_{m}/\delta_{t}-1}\right)}^{2}-{\left(0.5\delta_{t}\right)}^{2}}$$

(4) $$a=e+a_{0}$$

where *δ_m_* is the crack opening width, mm. *δ_t_* is the crack tip opening width, mm.

#### 2.4.2. Fracture Energy

Fracture energy (*G_F_*) is a parameter derived from the virtual crack model, accounting for concrete softening and reflecting the bending toughness of concrete. According to the load–deflection curve obtained from the notched three-point bending test, *G_F_* of steel-fiber-reinforced HSSCC was calculated using Formula (5).

(5) $$G_{F}=\frac{W_{0}+\mathrm{mg}\delta_{0}}{b\left(h-a_{0}\right)}$$

where *W*_0_ is defined as the area under the load–deflection curve, N·m. The range starts from 0 and ends at $\delta_{0}$, the deflection at the point of failure, where the specimen loses its load-bearing capacity completely. This accounts for all energy contributions prior to fracture. *m* is the mass of the specimens between two supports, kg. *g* is the gravitational acceleration with a value of 9.8 N/kg. *δ*_0_ is the deflection at the point of failure, mm. *b* and *h* are the width and height of the specimens, respectively, mm. *a*_0_ is the notch depth, mm.

## 3. Results and Discussion

### 3.1. Physical and Mechanical Properties of Steel-Fiber-Reinforced HSSCC

#### 3.1.1. Workability

**Figure 3:**
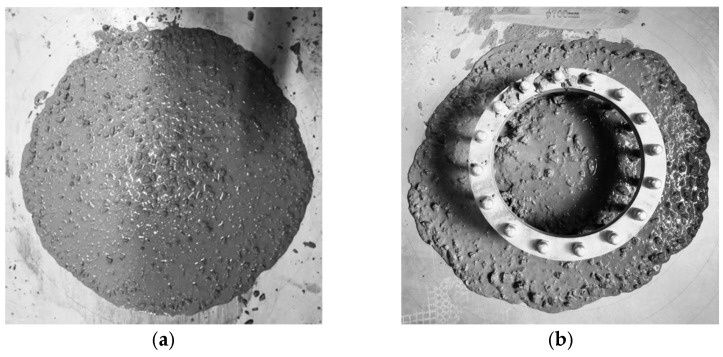
Workability of fresh mixed steel-fiber-reinforced HSSCC: (**a**) slump extension and T_500_ and (**b**) J-ring extension.

**Figure 4:**
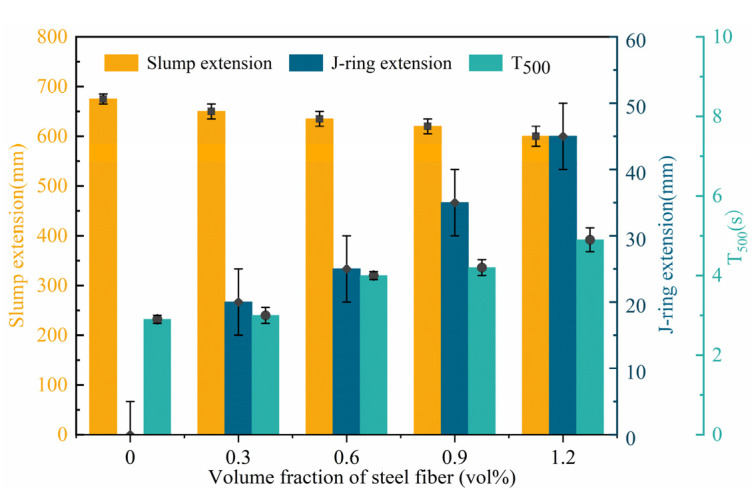
Workability of steel-fiber-reinforced HSSCC.

Figure 3 displays the on-site test images of workability of HSSCC, including slump extension, J-ring extension, and T_500_. As shown in Figure 4, both the slump extension and J-ring extension of HSSCC decreased gradually with increasing *V_f_*, while T_500_ increased. All steel-fiber-reinforced HSSCC mixtures met the workability requirements specified by the Chinese standard JGJ/T283-2012 [20]. The benchmark mixture HSSCC-0 exhibited a slump extension of 675 mm and a J-ring extension of 0 mm, with a T_500_ of 2.9 s. In comparison, the slump extension of HSSCC-1.2 decreased to 600 mm, and its J-ring extension and T_500_ increased to 45 mm and 4.9 s, respectively. These results indicated that the steel fibers with a high-aspect-ratio deteriorated the rheological properties of HSSCC mixtures, reducing workability with an increasing *V_f_*.

#### 3.1.2. Mechanical Properties of Steel-Fiber-Reinforced HSSCC

**Figure 5:**
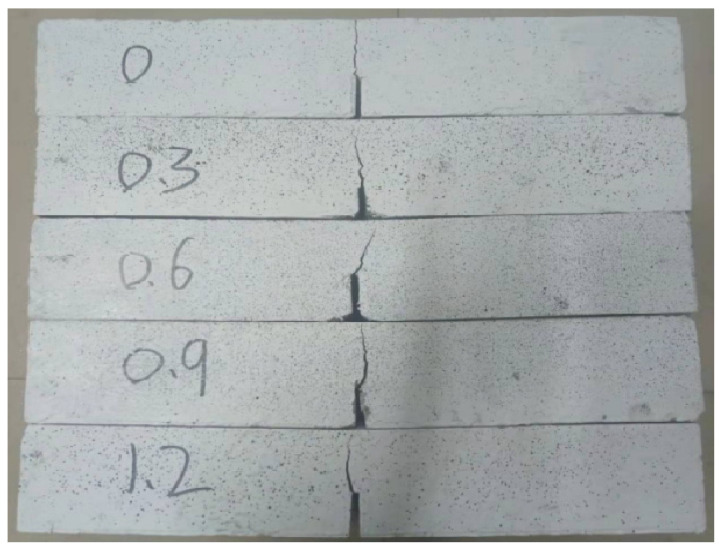
Failure modes of notched three-point bending beams.

**Figure 6:**
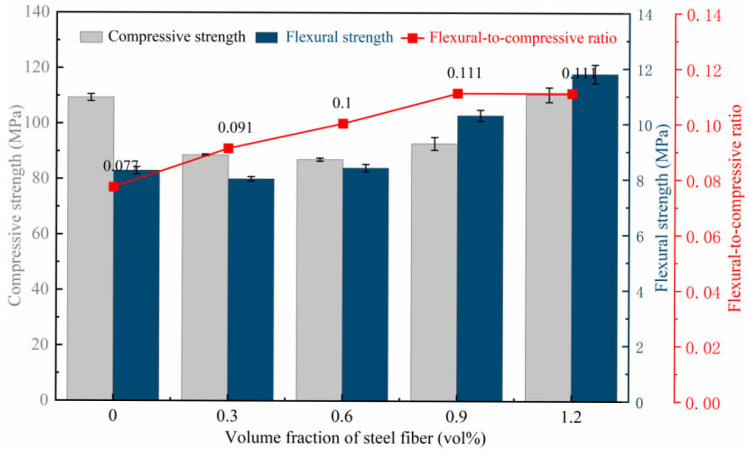
Mechanical properties of steel-fiber-reinforced HSSCC.

Figure 5 shows failure modes of notched three-point bending beams. As illustrated in Figure 6, the compressive strength and flexural strength of steel-fiber-reinforced HSSCC cured for 28 days exhibited a general trend of first decreasing and then increasing with the increase in *V_f_*. The compressive strength decreased to 87.0 MPa at a *V_f_* of 0.6%, while the flexural strength reached the lowest at a *V_f_* of 0.3%. Concurrently, the flexural-to-compressive ratio significantly enhanced with the increasing volume fraction of steel fiber. The compressive and flexural strength of HSSCC-1.2 cured for 28 days reached 110.5 MPa and 11.8 MPa, respectively, representing improvements of 1.1% and 42.2% compared to HSSCC-0. At lower *V_f_*, steel fibers were three-dimensional random distribution in the matrix, and new interfacial zones formed among fibers and matrix, which slightly deteriorated its mechanical properties. The interlocking degree among steel fibers improved as *V_f_* increased, forming an interconnected fiber network that enhanced the mechanical properties of HSSCC, especially its flexural and tensile properties. The compressive strength of steel-fiber-reinforced HSSCC cured for 28 days met all the C80 high-strength concrete requirements.

### 3.2. Fracture Mechanical Properties of Steel-Fiber-Reinforced HSSCC

#### 3.2.1. F-CMOD Curve Characteristics

**Figure 7:**
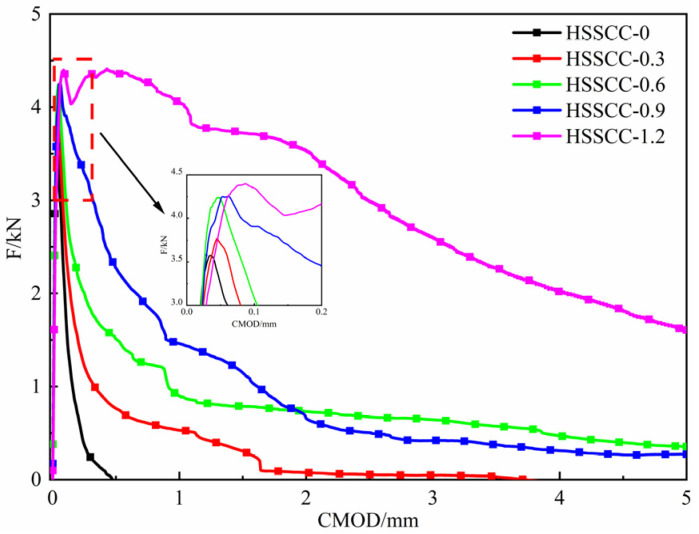
F-CMOD curves of steel-fiber-reinforced HSSCC.

Figure 7 presents the F-CMOD curves of steel-fiber-reinforced HSSCC, which are characterized by distinct elastic, stable crack propagation, and unstable failure stage. The F-CMOD curves evolved from a sharp peak to a steamed-bread peak as *V_f_* increased, accompanied by an increase in peak load at the corresponding peak CMOD. During the early unstable failure stage, higher *V_f_* led to greater CMOD of HSSCC under the same load and improved the load-bearing capacity at the same CMOD, providing an enhancement in fullness of the F-CMOD curves.

#### 3.2.2. Fracture Energy and Fracture Toughness

Table 5 presents the testing results of fracture parameters of steel-fiber-reinforced HSSCC. The incorporation of steel fibers significantly enhanced the *F_P_* and *K_IC_* of HSSCC. When *V_f_* was 1.2%, *F_P_* and *K_IC_* of steel-fiber-reinforced HSSCC reached their maximum values, representing increases of 23.5% and 45.4%, respectively, compared to the reference group. The CMOD at the peak load (CMOD_P_) reflected the ductility of the steel-fiber-reinforced HSSCC under flexural loading. As shown in Table 5, CMOD_P_ increased with rising *V_f_* and reached its maximum at a *V_f_* of 1.2%. Compared to HSSCC-0, the increase in CMOD_P_ of HSSCC-0.3, HSSCC-0.6, HSSCC-0.9, and HSSCC-1.2 was 27.3%, 28.5%, 73.8%, and 1110.7%, respectively.

The *G_F_* of the steel fiber-reinforced HSSCC also increased with the rise in *V_f_*, reaching its peak at *V_f_* of 1.2%, which was about 20 times higher than that of HSSCC-0. The significant enhancements in CMOD_P_ and *G_F_* for HSSCC-1.2 were attributed to the improvement in distribution density of steel fibers, which increased the number of fibers in the crack propagation tip region. The steel-fiber network bore parts of the flexural loads and underwent elastic deformation, and simultaneously enhanced the deformation capacity of HSSCC and mechanical energy dissipation during fracture. Consequently, the increase in CMOD_P_ and *G_F_* for HSSCC-1.2 was relatively significant.

### 3.3. DIC-Based Fracture Process Analysis

#### 3.3.1. Observation Segment Selection

**Figure 8:**
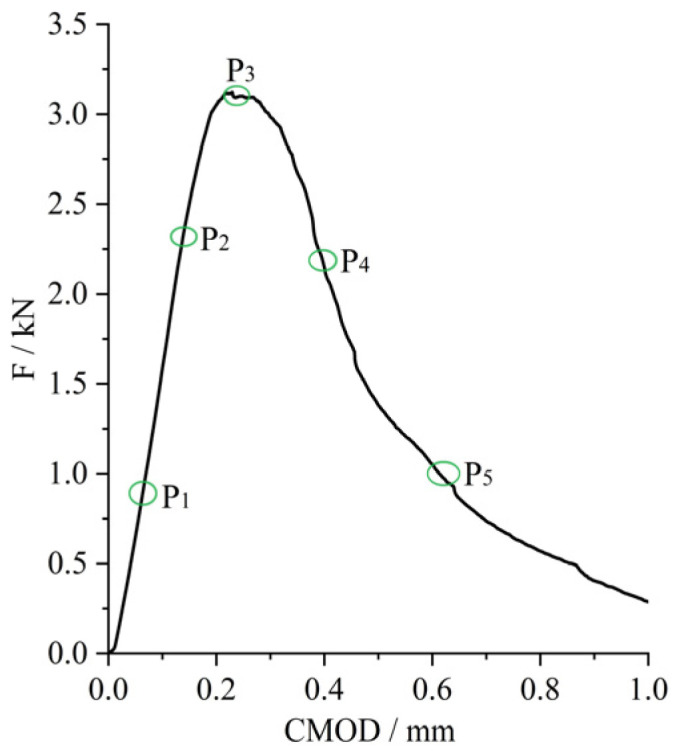
Illustration of observation segments selected for fracture process.

The schematic diagram of the observation points for the DIC analysis is shown in Figure 8. Based on the characteristics of the F-CMOD curve, no visible cracks appeared in the early loading stage of steel-fiber-reinforced HSSCC, which exhibited an approximately linear relationship between load and CMOD. Observation points P_1_ and P_2_ were selected for DIC analysis, corresponding to 30–40% and 65–75% of the peak load, respectively. With increasing load, microcracks appeared, and then, the fracture process zone formed in steel-fiber-reinforced HSSCC. During this stage, the CMOD increased rapidly; thus, the F-CMOD curves demonstrated a nonlinear trend. Due to the relatively short duration of this stage, the peak load, P_3_, was utilized as the observation point for DIC analysis. In the subsequent load softening stage, cracks in the notched beams propagated rapidly accompanied by a significant growth in the CMOD, while the corresponding load-bearing capacity reduced until complete loss. In this stage, points P_4_ and P_5_ were selected for DIC analysis, corresponding to 80–90% and 50–60% of the peak load, respectively.

#### 3.3.2. Horizontal Displacement Cloud

**Figure 9:**
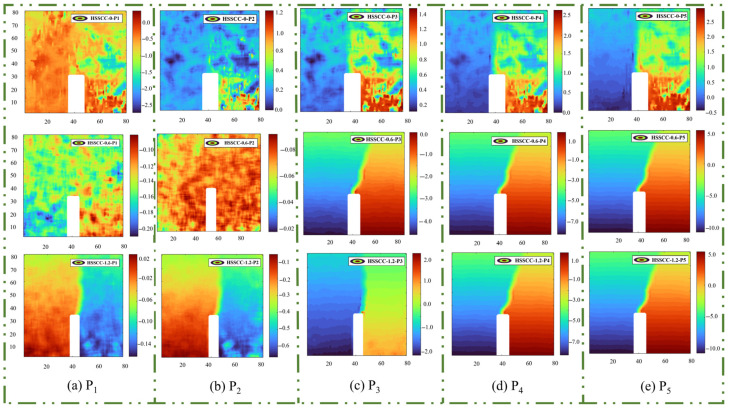
Horizontal displacement contours of steel-fiber-reinforced HSSCC at varied observation segments: (**a**) Observation Point P_1_ of the fracture process; (**b**) Observation Point P_2_ of the fracture process; (**c**) Observation Point P_3_ of the fracture process; (**d**) Observation Point P_4_ of the fracture process; (**e**) Observation Point P_5_ of the fracture process.

At the early loading stage, the stress state exhibited minimal upward compression and downward tension. Owing to the non-uniformity and internal defects of steel-fiber-reinforced HSSCC, as shown in Figure 9a, the displacement cloud presented a point-like distribution at this stage, during which the stress and displacement at each point of the notched beam varied unevenly. As the load continued to increase, as indicated in Figure 9b, the positive or negative displacement zone in the lower part of the notched beam expanded upward, manifesting the occurrence of macroscopic cracks at the tip of the notch. When the load reached its peak, Figure 9c showed that a notched beam did not immediately destroy after cracking. Concurrently, the displacement mutation zone propagated further upward, forming a distinct boundary line, while the macroscopic crack developed steadily. Subsequently, the load-carrying capacity of the HSSCC began to decline in the load softening stage. As described in Figure 9d,e, the horizontal displacement increased rapidly as the macroscopic crack destabilized, leading to the formation of through-cracks until the complete failure of the specimens [23].

As can be observed from the first line of Figure 9, the displacement mutation zone was not formed at the crack tip region throughout the fracture process of HSSCC-0. Instead, unstable crack failure occurred in the lower right portion of the specimen within the areas covered by P_3_, P_4_, and P_5_. In the fracture process of HSSCC-1.2, a tensile pressure demarcation line, corresponding to the displacement mutation zone, was observed at the crack tip region near the observation point P_2_. As the crack propagated through the notch region, a tension–compression stress transition zone occurred at the observation point P_3_ and even at P_2_, indicating that the three-dimensional overlapping steel-fiber network altered the crack propagation path.

#### 3.3.3. Horizontal Strain Cloud

**Figure 10:**
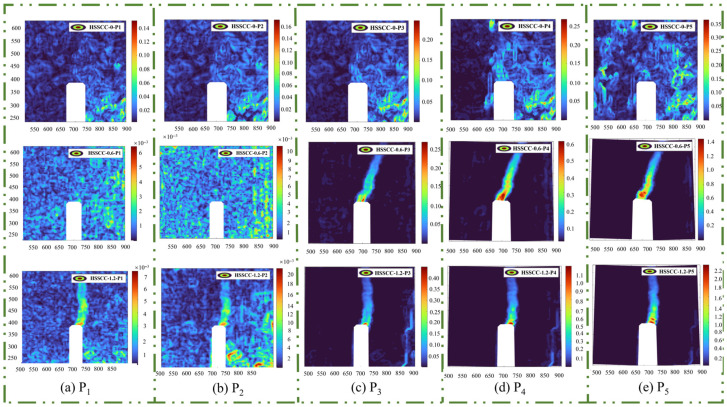
Horizontal strain cloud of steel-fiber-reinforced HSSCC at varied observation segments: (**a**) Observation Point P1 of the fracture process; (**b**) Observation Point P2 of the fracture process; (**c**) Observation Point P3 of the fracture process; (**d**) Observation Point P4 of the fracture process; (**e**) Observation Point P5 of the fracture process.

Corresponding to the horizontal displacement cloud, the horizontal strain cloud also exhibited significant variation across the observation regions. As illustrated in Figure 10a, during the initial loading stage, the horizontal strain cloud exhibited alternating blue-green stripes or punctate distributions. The strain fringes appeared in an alternating and cyclic pattern as the load increased, during which specimens experienced microcrack dispersion and stress redistribution [24]. As depicted in Figure 10b, the horizontal strain increased with the applied load. Moreover, the fracture process zone was observed to initiate at the tip of the notched beams in both HSSCC-0.6 and SCC-1.2 specimens.

Figure 10c presents the horizontal strain cloud of HSSCC under the peak load. The fracture process zones of HSSCC-0.6 and HSSCC-1.2 extended further in the upward direction, resulting in an increase in horizontal strain. Consequently, the load-bearing capacity of the notched beams approached their ultimate limit. In contrast, no obvious fracture process zone was observed in HSSCC-0, indicating that HSSCC without steel fibers failed without significant horizontal strain at the tip of the notched beam under the action of bending load. As illustrated in Figure 10d,e, specimens experienced unstable failure upon reaching the peak load, while HSSCC-0.6 and HSSCC-1.2 demonstrated greater ductility without immediate brittle fracture. This observation aligns with previous studies, which have concluded that steel fibers can effectively delay crack propagation [12].

Based on the fiber–matrix interaction model, steel fibers in the fracture process zone spanned both sides of cracks via the bridging mechanism, thereby transferring stress through the bonding force at the fiber–matrix interface. Furthermore, they delayed the propagation path of the crack tip through the crack-arresting mechanism. These two mechanisms worked synergistically to continuously dissipate the energy introduced by external loads during crack development, markedly improving the fracture resistance of concrete [25].

### 3.4. Fracture Behavior of Steel-Fiber-Reinforced HSSCC-Based DIC Analysis

Based on the DIC analysis of the horizontal displacement and horizontal strain cloud presented in Figure 9 and Figure 10 for steel-fiber-reinforced HSSCC, it was evident that the notched beam specimens experienced three stages in the fracture process: the initiation of dispersed microcracks, stable crack propagation, and unstable crack failure. In the initial loading stage, the stress level of overall specimens and the notched region changed in response to variations in the load they bore. Concurrently, the microcracks originated from the pre-existing defects or pores within specimens propagated progressively. As the load continued increasing, although macroscopic cracks initiated at the tip of the incision and progressively propagated upward, the bearing capacity of steel-fiber-reinforced HSSCC kept rising, which was characterized as the crack stable propagation stage. When the load reached the peak, the load-bearing capacity of the specimens began to decrease, and the macroscopic cracks extended unstably until complete fracture occurred, which was the unstable crack failure stage.

By conducting a comparative analysis of three vertically arranged images presented in Figure 9 and Figure 10, it was demonstrated that the horizontal displacement and horizontal strain of the steel-fiber-reinforced HSSCC increased with a rise in *V_f_* of steel fibers. As the crack tip extended to the steel-fiber-reinforced regions, cracks either bypassed or traversed the uniformly distributed steel fibers, thereby consuming additional mechanical energy. Therefore, the strain level of the steel-fiber-reinforced HSSCC correspondingly elevated.

Figure 9a,b and Figure 10a,b illustrate the microcrack dispersion and initiation stage, indicating a slight enhancement in the horizontal displacement and strain of HSSCC, caused by the increase in the number of initiated microcracks with the rise in steel fiber distribution concentration. Moreover, the fracture process zone generated at the notch tip of HSSCC-1.2 notably increased the consumption of mechanical energy during the fraction test. In the stable crack propagation stage, as depicted in Figure 9c and Figure 10c, no distinct fracture process zone appeared at the notch tip of HSSCC-0 under peak load. Consequently, the horizontal displacement and strain of HSSCC-0 were lower than those of steel-fiber-reinforced HSSCC at their crack initiation stage, and thus, the CMOD_P_ of HSSCC-0 was significantly less than of HSSCC-0.6 and HSSCC-1.2.

The combined analysis of Figure 7 and Figure 9d,e and Figure 10d,e reveals that the notch tip of HSSCC-0 experienced no obvious horizontal displacement and horizontal strain during the unstable crack failure stage, which implied that it rapidly lost its load-bearing capacity. In contrast, HSSCC-0.6 and HSSCC-1.2 exhibited progressive horizontal displacement and strain at the notch tip as their load-bearing capacity declined. When crack tips propagated into steel fiber-reinforced regions, steel fibers underwent tensile deformation. An increase in *V_f_* enhanced the load-bearing capacity at the same CMOD for HSSCC, and thus, both fracture toughness and fracture energy were significantly improved [12].

## 4. Conclusions

On the basis of investigating the physical and mechanical properties of steel-fiber-reinforced high-strength self-compacting concrete (HSSCC), the influence of steel fibers on its fracture characteristics was clarified through the three-point bending tests with notched beams. By integrating the digital image correlation (DIC) technique, the fracture process of steel-fiber-reinforced HSSCC was analyzed to clarify the improvement mechanism of steel fibers on its fracture toughness. The main conclusions are drawn as follows:

1. The incorporation of steel fibers obviously improved the flexural properties of HSSCC and deteriorated its workability. As *V_f_* of steel fibers increased, the 28-day compressive strength and flexural strength of HSSCC exhibited an initial decrease and then an enhancement, and reached their maximum values of 110.5 MPa and 11.8 MPa at *V_f_* of 1.2%, respectively, whereas the flexural-to-compressive ratio nearly linearly increased. The workability and compressive strength of HSSCC met the requirements of the relevant specifications for SCC and high-strength concrete of Grade C80.
2. The fracture mechanics characteristics of HSSCC enhanced with an increase in *V_f_* of steel fibers. When *V_f_* was 1.2%, the peak load (*F_P_*), peak opening displacement (CMOD_P_), fracture toughness (*K_IC_*), and fracture energy (*G_F_*) of steel-fiber-reinforced HSSCC increased by 23.5%, 45.4%, 11.1 times, and 20.1 times compared with those of HSSCC-0, respectively. Steel fibers significantly improved the load-bearing capacity and fracture toughness of HSSCC under flexural loading through the effects of crack bridging and energy dissipation.
3. The deformation behavior during the fracture process of HSSCC was revealed by the DIC technique. In the case of HSSCC-0, the mutation zone of the horizontal displacement clouds did not form during the whole fracture, and the fracture process zones of horizontal strain clouds came into being at the load softening stage, whereas both of these zones of HSSCC-0.6 and HSSCC-1.2 occurred at the peak loading and the early loading stage, respectively. During the fracture of steel-fiber-reinforced HSSCC, both the horizontal deformation and horizontal strain magnified significantly with an increase in *V_f_*, indicating that the increased concentration of steel fibers effectively delayed the overlap and expansion of the cracks at the tip of notched beams.
4. This study integrated the DIC technology to unveil the deformation behavior of HSSCC during its fracture process and the toughening mechanism of steel fibers. In future studies, X-ray computed tomography will be employed to analyze the distribution state of steel fibers, aiming to disclose the correlation between crack orientations and their crack-arresting effects. Additionally, further investigations will be carried out to explore the degradation law of the flexural tensile deformation behavior of steel-fiber-reinforced HSSCC under freeze–thaw cycles. This endeavor is expected to provide essential data for the promotion of its engineering applications.

## Figures and Tables

**Table 1:** Technical indexes of ordinary Portland cement.

Setting Time/Min		Compressive Strength/MPa		Flexural Strength/MPa	
Initial	Final	3d	28d	3d	28d
194	271	30.1	59.6	7.2	9.8

**Table 2:** Chemical components of slag.

	SiO_2_	Al_2_O_3_	Fe_2_O_3_	CaO	K_2_O	MgO	Na_2_O	TiO_2_	Others
Content/wt%	29.73	13.46	1.08	42.38	0.73	6.76	0.49	1.56	3.81

**Table 3:** Technical specifications of steel fiber.

Aspect Ratio	Length	Elasticity Modulus	Tensile Strength
63	35 ± 3 mm	220 GPa	≥380 MPa

**Table 4:** Mixture of steel-fiber-reinforced HSSCC (kg/m^3^).

Code	Cement	Slag	Silica Fume	Steel Fiber	Water	River Sand	Gravel	Water Reducer
HSSCC-0	435.6	121.0	48.4	-	193.6	710.4	941.0	1.5%
HSSCC-0.3	435.6	121.0	48.4	23.6	193.6	707.7	938.1	1.5%
HSSCC-0.6	435.6	121.0	48.4	47.2	193.6	704.2	933.7	1.5%
HSSCC-0.9	435.6	121.0	48.4	70.8	193.6	700.7	929.3	2.0%
HSSCC-1.2	435.6	121.0	48.4	94.4	193.6	697.2	924.9	2.0%

Note: the “0.3” of HSSCC-0.3 refers to the volume fraction of steel fibers, and so on after that.

**Table 5:** Fracture parameters of steel-fiber-reinforced HSSCC.

Code	*F_P_*		*K_IC_*		CMOD		*G_F_*	
	Value (kN)	Increase (%)	Value (kPa·m^1/2^)	Increase (%)	Value (μm)	Increase (%)	Value (N·m^−1^)	Increase (%)
HSSCC-0	3.6	-	894.6	-	35.5	-	121.5	-
HSSCC-0.3	3.8	5.6	958.0	7.1	45.2	27.3	316.3	160.3
HSSCC-0.6	4.2	16.7	1080.5	20.8	45.6	28.5	823.6	578.0
HSSCC-0.9	4.3	19.4	1089.5	21.8	61.7	73.8	922.0	660.5
HSSCC-1.2	4.4	22.2	1300.4	45.4	429.8	1110.6	2567.8	2013.4

## Data Availability

The original contributions presented in this study are included in the article. Further inquiries can be directed to the corresponding authors.
